# Supplementary material for: Autoinhibition and regulation by phosphoinositides of ATP8B1, a human lipid flippase associated with intrahepatic cholestatic disorders
Source: eLife. 2022 Apr 13;11:e75272. doi: 10.7554/eLife.75272 (PMC9045818; doi:10.7554/eLife.75272)
Supplement: Figure 1—figure supplement 1—source data 1. [file elife-75272-fig1-figsupp1-data1.pdf]

Figure 1 – figure supplement 1E – source data

Table format: Grouped		Group A<br>normalized to the number of W	
		A:Y1	A:Y2
1	ATP8B1	0.387	0.362
2	CDC50A	0.343	0.420
3	Title		
