# Supplementary material for: Autoinhibition and regulation by phosphoinositides of ATP8B1, a human lipid flippase associated with intrahepatic cholestatic disorders
Source: eLife. 2022 Apr 13;11:e75272. doi: 10.7554/eLife.75272 (PMC9045818; doi:10.7554/eLife.75272)
Supplement: Figure 4—figure supplement 3—source data 1. [file elife-75272-fig4-figsupp3-data1.pdf]

Figure 4 – figure supplement 3B – source data

Table format: Grouped	Group A														
	WT												D454N		
	A:Y1	A:Y2	A:Y3	A:Y4	A:Y5	A:Y6	A:Y7	A:Y8	A:Y9	A:Y10	A:Y11	A:Y12	B:Y1	B:Y2	B:Y3
1 DDM/CHS	0.154	0.152	0.157	0.125	0.119	0.127	0.114	0.113	0.110	0.101	0.107	0.113	0.195	0.146	0.182
2 +POPC	0.176	0.171	0.161										0.202	0.194	0.176
3 +PI(4,5)P2	0.126	0.121	0.116										0.135	0.115	0.122
4 +trypsin	0.784	0.743	0.758	0.770	0.750	0.758	0.697	0.707	0.666	0.584	0.508	0.509	0.143	0.142	0.126
5 +BeFx	0.147	0.145	0.152	0.157	0.137	0.149	0.134	0.119	0.151	0.150	0.156	0.134	0.149	0.120	0.115
6 Title															
