# Supplementary material for: Autoinhibition and regulation by phosphoinositides of ATP8B1, a human lipid flippase associated with intrahepatic cholestatic disorders
Source: eLife. 2022 Apr 13;11:e75272. doi: 10.7554/eLife.75272 (PMC9045818; doi:10.7554/eLife.75272)
Supplement: Figure 4—figure supplement 4—source data 1. [file elife-75272-fig4-figsupp4-data1.pdf]

Figure 4 – figure supplement 4A – source data

		X	Group A		
		log[BeF], M	Data Set-A		
		X	A:Y1	A:Y2	A:Y3
1	Titl	-6.300000	103.021100	104.717000	96.530790
2	Titl	-6.000000	102.014100	100.943400	89.592370
3	Titl	-5.300000	96.978850	102.830200	93.061580
4	Titl	-5.000000	107.049300	98.113200	93.061580
5	Titl	-4.700000	100.000000	74.528310	87.857770
6	Titl	-4.300000	52.870090	45.283020	43.625320
7	Titl	-4.000000	7.754280	9.433962	8.065915
8	Titl	-3.320000	-5.236657	-0.943396	5.464007
9	Titl	-3.020000	-2.819738	-3.301887	-8.846487

Figure 4 – figure supplement 4A – fit

	Nonlin fit<br>Table of results	A<br>Data Set-A
		Y
1	**log(inhibitor) vs. response -- Variable slope (four parameters)**	
2	**Best-fit values**	
3	Bottom	-3.284
4	Top	99.05
5	LogIC50	-4.314
6	HillSlope	-2.599
7	IC50	4.855e-005
8	Span	102.3
9	**95% CI (profile likelihood)**	
10	Bottom	-8.399 to 1.698
11	Top	95.29 to 103.0
12	LogIC50	-4.367 to -4.265
13	HillSlope	-3.686 to -1.994
14	IC50	4.291e-005 to 5.434e-005
15	**Goodness of Fit**	
16	Degrees of Freedom	23
17	R squared	0.9842
18	Sum of Squares	842.3
19	Sy.x	6.051
20		
21	**Number of points**	
22	# of X values	27
23	# Y values analyzed	27

Figure 4 – figure supplement 4B – source data

		X	Group A		
		ATP	specific activity		
		X	A:Y1	A:Y2	A:Y3
1	Title	0.000000	-0.026916	-0.001737	0.018234
2	Title	0.999001	-0.000868	0.075539	0.048623
3	Title	4.975124	0.078144	0.059910	0.125898
4	Title	19.870840	0.200569	0.254402	0.244851
5	Title	49.529470	0.401138	0.399402	0.377695
6	Title	98.570720	0.505330	0.500121	0.499252
7	Title	491.776400	0.636438	0.699822	0.685061
8	Title	980.873000	0.685929	0.738894	0.738894
9	Title	4799.744000	0.743235	0.682456	0.707636

Figure 4 – figure supplement 4B – fit

	Nonlin fit<br>Table of results	A<br>specific activity
		Y
1	**Michaelis-Menten**	
2	**Best-fit values**	
3	Vmax	0.7316
4	Km	42.47
5	**Std. Error**	
6	Vmax	0.01035
7	Km	2.784
8	**95% CI (asymptotic)**	
9	Vmax	0.7102 to 0.7529
10	Km	36.73 to 48.20
11	**Goodness of Fit**	
12	Degrees of Freedom	25
13	R squared	0.9912
14	Sum of Squares	0.01857
15	Sy.x	0.02725
16	**Constraints**	
17	Km	Km > 0
18		
19	**Number of points**	
20	# of X values	27
21	# Y values analyzed	27
