# Supplementary material for: Autoinhibition and regulation by phosphoinositides of ATP8B1, a human lipid flippase associated with intrahepatic cholestatic disorders
Source: eLife. 2022 Apr 13;11:e75272. doi: 10.7554/eLife.75272 (PMC9045818; doi:10.7554/eLife.75272)
Supplement: Figure 5—source data 1. [file elife-75272-fig5-data1.pdf]

Figure 5B – source data 1

		X	Group A			Group B		
		log[peptide], M	C-ter			Phosphorylated C-ter		
		X	A:Y1	A:Y2	A:Y3	B:Y1	B:Y2	B:Y3
1	Title	-9.0000	96	101	99	104	98	99
2	Title	-8.5200	101	107	96	99	103	97
3	Title	-8.0000	98	97	96	109	96	100
4	Title	-7.5300	103	101	98	100	103	110
5	Title	-7.0000	104	104	97	94	99	104
OK CM210518_other fits		-6.5300	103	99	97	106	102	110
7	Title	-6.0110	103	95	99	107	102	112
8	Title	-5.5400	94	86	87	105	101	116
9	Title	-5.0200	74	63	69	108	100	108
10	Title	-4.5400	47	42	40	108	99	109
11	Title	-4.0400		21	22	94	82	100
12	Title	-3.7400			10		76	87
13	Title							

Figure 5B – fit

	Nonlin fit	A	B
		C-ter	Phosphorylated C-ter
		Y	Y
1	log(inhibitor) vs. normalized response -- Variable slope		
2	Best-fit values		
3	LogIC50	-4.655	-3.423
4	HillSlope	-1.013	-1.965
5	IC50	2.213e-005	0.0003774
6	Std. Error		
7	LogIC50	0.02429	0.2058
8	HillSlope	0.05552	1.057
9	95% Confidence Intervals		
10	LogIC50	-4.704 to -4.605	-3.842 to -3.005
11	HillSlope	-1.126 to -0.8996	-4.116 to 0.1858
12	IC50	1.975e-005 to 2.481e-005	0.0001439 to 0.000989
13	Goodness of Fit		
14	Degrees of Freedom	31	33
15	R square	0.9839	0.3462
16	Absolute Sum of Squares	396.1	1461
17	Sy.x	3.575	6.653
18			
19	Number of points		
20	Analyzed	33	35
21			
