# Supplementary material for: Autoinhibition and regulation by phosphoinositides of ATP8B1, a human lipid flippase associated with intrahepatic cholestatic disorders
Source: eLife. 2022 Apr 13;11:e75272. doi: 10.7554/eLife.75272 (PMC9045818; doi:10.7554/eLife.75272)
Supplement: Figure 5—source data 2. [file elife-75272-fig5-data2.pdf]

Figure 5C – source data

Table format: XY		X	Group A					Group B				
		log[peptide]	dC E1174 (C-ter)					dC E1174 (Phos C-ter)				
		X	A:Y1	A:Y2	A:Y3	A:Y4	A:Y5	B:Y1	B:Y2	B:Y3	B:Y4	B:Y5
1	Title	-9.0000	97	96	95	87		89	101			91
2	Title	-8.5240	93	106	83	86		91	81			82
3	Title	-8.0030	88	89	90	82		89	73	78	79	77
4	Title	-7.5270	74	64	81	68		77	77	84	65	77
5	Title	-7.0070	48	46	65	51		70	66	78	62	77
6	Title	-6.5310	28	36	39	34		65	58	69	58	75
7	Title	-6.0110	27	25	19	24		57	51	62	52	64
8	Title	-5.5350	18	17	13	21		45	41	49	43	60
9	Title	-5.0150				18		39	47	33	33	48
10	Title	-4.5440				17				27	21	33
11	Title	-4.0420								24	15	30
12	Title	-3.7440										
13	Title											

Figure 5C – fit C-ter peptide

	Nonlin fit	A<br>dC E1174 (C-ter)
		Y
1	log(inhibitor) vs. response -- Variable slope (four parameters)	
2	Best-fit values	
3	Bottom	15.76
4	Top	95.62
5	LogIC50	-7.091
6	HillSlope	-0.9289
7	IC50	8.116e-008
8	Span	79.86
9	Std. Error	
10	Bottom	2.894
11	Top	2.804
12	LogIC50	0.06927
13	HillSlope	0.1349
14	Span	4.709
15	95% Confidence Intervals	
16	Bottom	9.848 to 21.67
17	Top	89.90 to 101.3
18	LogIC50	-7.232 to -6.949
19	HillSlope	-1.204 to -0.6534
20	IC50	5.860e-008 to 1.124e-007
21	Span	70.25 to 89.48
22	Goodness of Fit	
23	Degrees of Freedom	30
24	R square	0.9697
25	Absolute Sum of Squares	963.6
26	Sy.x	5.667
27		
28	Number of points	
29	Analyzed	34

Figure 5C – fit phosphorylated C-ter peptide

	Nonlin fit	A<br>dC E1174 (Phos C-ter)
		Y
1	log(inhibitor) vs. normalized response -- Variable slope	
2	Best-fit values	
3	LogIC50	-5.708
4	HillSlope	-0.2911
5	IC50	1.957e-006
6	Std. Error	
7	LogIC50	0.07221
8	HillSlope	0.01743
9	95% Confidence Intervals	
10	LogIC50	-5.854 to -5.563
11	HillSlope	-0.3262 to -0.2560
12	IC50	1.400e-006 to 2.735e-006
13	Goodness of Fit	
14	Degrees of Freedom	45
15	R square	0.9107
16	Absolute Sum of Squares	1859
17	Sy.x	6.428
18		
19	Number of points	
20	Analyzed	47
