# Supplementary material for: Autoinhibition and regulation by phosphoinositides of ATP8B1, a human lipid flippase associated with intrahepatic cholestatic disorders
Source: eLife. 2022 Apr 13;11:e75272. doi: 10.7554/eLife.75272 (PMC9045818; doi:10.7554/eLife.75272)
Supplement: Figure 5—source data 3. [file elife-75272-fig5-data3.pdf]

Figure 5D – source data

Table format: Grouped		Group A			Group B		
		non phosphorylated C-ter peptide			phosphorylated C-ter peptide		
		Mean	SD	N	Mean	SD	N
1	ΔC	0.08100	0.01400	34	1.96000	0.35000	47

Table format: Grouped		Group A			Group B		
		non phosphorylated C-ter peptide			phosphorylated C-ter peptide		
		Mean	SD	N	Mean	SD	N
1	ΔNC	22.10000	1.20000	33	377.40000	227.00000	35
