# Supplementary material for: Autoinhibition and regulation by phosphoinositides of ATP8B1, a human lipid flippase associated with intrahepatic cholestatic disorders
Source: eLife. 2022 Apr 13;11:e75272. doi: 10.7554/eLife.75272 (PMC9045818; doi:10.7554/eLife.75272)
Supplement: Figure 5—source data 4. [file elife-75272-fig5-data4.pdf]

# Figure 5E – source data

| | Table format: Grouped | Group A | | | Group B | | | Group C | | |
|---|---|---|---|---|---|---|---|---|---|---|
| | | ATP8B1/CDC50A dNC | | | Drs2 dNC | | | Na/K | | |
| | | A:Y1 | A:Y2 | A:Y3 | B:Y1 | B:Y2 | B:Y3 | C:Y1 | C:Y2 | C:Y3 |
| 1 | -C-ter peptide | 100 | 100 | 100 | 98 | 99 | 103 | 106.7 | 97.8 | 95.4 |
| 2 | +C-ter peptide | 10 | | | 100 | 77 | 83 | 85.9 | 85.5 | 90.1 |
| 3 | Title | | | | | | | | | |

# Figure 5E – statistics

ANOVA results × | Multiple comparisons × | Narrative results ×

| | 2way ANOVA<br>ANOVA results | | | | | |
|---|---|---|---|---|---|---|
| 1 | Table Analyzed | % of control, grouped | | | | |
| 2 | | | | | | |
| 3 | **Two-way ANOVA** | Ordinary | | | | |
| 4 | Alpha | 0.05 | | | | |
| 5 | | | | | | |
| 6 | **Source of Variation** | **% of total variation** | **P value** | **P value summary** | **Significant?** | |
| 7 | Interaction | 45.92 | <0.0001 | **** | Yes | |
| 8 | C-ter peptide | 65.40 | <0.0001 | **** | Yes | |
| 9 | Protein type | 45.88 | <0.0001 | **** | Yes | |
| 10 | | | | | | |
| 11 | **ANOVA table** | **SS (Type III)** | **DF** | **MS** | **F (DFn, DFd)** | **P value** |
| 12 | Interaction | 3551 | 2 | 1776 | F (2, 10) = 46.42 | P<0.0001 |
| 13 | C-ter peptide | 5058 | 1 | 5058 | F (1, 10) = 132.2 | P<0.0001 |
| 14 | Protein type | 3548 | 2 | 1774 | F (2, 10) = 46.38 | P<0.0001 |
| 15 | Residual | 382.5 | 10 | 38.25 | | |
| 16 | | | | | | |
| 17 | **Difference between row means** | | | | | |
| 18 | Predicted (LS) mean of -C-ter peptide | 99.99 | | | | |
| 19 | Predicted (LS) mean of +C-ter peptide | 61.28 | | | | |
| 20 | Difference between predicted means | 38.71 | | | | |
| 21 | SE of difference | 3.367 | | | | |
| 22 | 95% CI of difference | 31.21 to 46.21 | | | | |
| 23 | | | | | | |
| 24 | **Data summary** | | | | | |
| 25 | Number of columns (Protein type) | 3 | | | | |
| 26 | Number of rows (C-ter peptide) | 2 | | | | |
| 27 | Number of values | 16 | | | | |
| 28 | | | | | | |
