# Supplementary figures and images for: Autoinhibition and regulation by phosphoinositides of ATP8B1, a human lipid flippase associated with intrahepatic cholestatic disorders
Source: eLife. 2022 Apr 13;11:e75272. doi: 10.7554/eLife.75272 (PMC9045818; doi:10.7554/eLife.75272)

Figure 5 – figure supplement 1C – source data

| 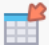 | Group A | Group B             |
|-----------------------------------------------------------------------------------|---------|---------------------|
|                                                                                   | none    | 70 µM C-ter peptide |
|                                                                                   |         |                     |
| 1                                                                                 | 0.064   | 0.068               |
| 2                                                                                 | 0.069   | 0.063               |
| 3                                                                                 | 0.065   | 0.064               |
| .                                                                                 |         |                     |

Supplement: Figure 5—figure supplement 1—source data 1. [file elife-75272-fig5-figsupp1-data1.pdf]
