# Supplementary material for: Autoinhibition and regulation by phosphoinositides of ATP8B1, a human lipid flippase associated with intrahepatic cholestatic disorders
Source: eLife. 2022 Apr 13;11:e75272. doi: 10.7554/eLife.75272 (PMC9045818; doi:10.7554/eLife.75272)
Supplement: Figure 6—source data 1. [file elife-75272-fig6-data1.pdf]

Figure 6A – source data

	Group A	Group B	Group C	Group D	Group E	Group F	Group G	Group H
	PC	PS	PE	Lyso-PC	Edelfosine	Miltefosine	CL	SM
1	94.5	50.3	142.9	33.5	20.8	18.6	12.7	10.00
2	103.9	48.9	136.2	39.1	25.4	22.9	9.9	12.90
3	101.6	48.5	144.5	36.6	19.1	16.5	8.6	11.00
4								

Figure 6A – tabular results – SM vs PC

	Unpaired t test<br>Tabular results	
1	Table Analyzed	screen lipids + drugs_for new figure 5A
2		
3	Column H	SM
4	vs.	vs.
5	Column A	PC
6		
7	**Unpaired t test**	
8	P value	<0.0001
9	P value summary	****
10	Significantly different (P < 0.05)?	Yes
11	One- or two-tailed P value?	Two-tailed
12	t, df	t=30.03, df=4
13		
14	**How big is the difference?**	
15	Mean of column A	100.0
16	Mean of column H	11.30
17	Difference between means (H - A) ± SEM	-88.70 ± 2.954
18	95% confidence interval	-96.90 to -80.50
19	R squared (eta squared)	0.9956
20		
21	**F test to compare variances**	
22	F, DFn, Dfd	11.06, 2, 2
23	P value	0.1658
24	P value summary	ns
25	Significantly different (P < 0.05)?	No
26		
27	**Data analyzed**	
28	Sample size, column A	3
29	Sample size, column H	3

Figure 6A – descriptive statistics – SM vs PC

Unpaired t test<br>Descriptive statistics		PC	SM
1	Number of values	3	3
2			
3	Minimum	94.50	10.00
4	25% Percentile	94.50	10.00
5	Median	101.6	11.00
6	75% Percentile	103.9	12.90
7	Maximum	103.9	12.90
8			
9	Mean	100.0	11.30
10	Std. Deviation	4.900	1.473
11	Std. Error of Mean	2.829	0.8505
12			
13	Lower 95% CI	87.83	7.641
14	Upper 95% CI	112.2	14.96

Figure 6A – tabular results – SM vs PE

Unpaired t test<br>Tabular results		
1	Table Analyzed	screen lipids + drugs_for new figure 5A
2		
3	Column H	SM
4	vs.	vs.
5	Column C	PE
6		
7	**Unpaired t test**	
8	P value	<0.0001
9	P value summary	****
10	Significantly different (P < 0.05)?	Yes
11	One- or two-tailed P value?	Two-tailed
12	t, df	t=48.46, df=4
13		
14	**How big is the difference?**	
15	Mean of column C	141.2
16	Mean of column H	11.30
17	Difference between means (H - C) ± SEM	-129.9 ± 2.681
18	95% confidence interval	-137.3 to -122.5
19	R squared (eta squared)	0.9983
20		
21	**F test to compare variances**	
22	F, DFn, Dfd	8.935, 2, 2
23	P value	0.2013
24	P value summary	ns
25	Significantly different (P < 0.05)?	No
26		
27	**Data analyzed**	
28	Sample size, column C	3
29	Sample size, column H	3

Figure 6A – descriptive statistics – SM vs PE

Tabular results<br>Unpaired t test<br>Descriptive statistics		PE	SM
1	Number of values	3	3
2			
3	Minimum	136.2	10.00
4	25% Percentile	136.2	10.00
5	Median	142.9	11.00
6	75% Percentile	144.5	12.90
7	Maximum	144.5	12.90
8			
9	Mean	141.2	11.30
10	Std. Deviation	4.403	1.473
11	Std. Error of Mean	2.542	0.8505
12			
13	Lower 95% CI	130.3	7.641
14	Upper 95% CI	152.1	14.96

Figure 6A – tabular results – SM vs PS

	Unpaired t test<br>Tabular results	
1	Table Analyzed	screen lipids + drugs_for new figure 5A
2		
3	Column H	SM
4	vs.	vs.
5	Column B	PS
6		
7	**Unpaired t test**	
8	P value	<0.0001
9	P value summary	****
10	Significantly different (P < 0.05)?	Yes
11	One- or two-tailed P value?	Two-tailed
12	t, df	t=37.54, df=4
13		
14	**How big is the difference?**	
15	Mean of column B	49.23
16	Mean of column H	11.30
17	Difference between means (H - B) ± SEM	-37.93 ± 1.011
18	95% confidence interval	-40.74 to -35.13
19	R squared (eta squared)	0.9972
20		
21	**F test to compare variances**	
22	F, DFn, Dfd	2.429, 2, 2
23	P value	0.5832
24	P value summary	ns
25	Significantly different (P < 0.05)?	No
26		
27	**Data analyzed**	
28	Sample size, column B	3
29	Sample size, column H	3

Figure 6A – descriptive statistics – SM vs PS

	Unpaired t test<br>Descriptive statistics	PS	SM
1	Number of values	3	3
2			
3	Minimum	48.50	10.00
4	25% Percentile	48.50	10.00
5	Median	48.90	11.00
6	75% Percentile	50.30	12.90
7	Maximum	50.30	12.90
8			
9	Mean	49.23	11.30
10	Std. Deviation	0.9452	1.473
11	Std. Error of Mean	0.5457	0.8505
12			
13	Lower 95% CI	46.89	7.641
14	Upper 95% CI	51.58	14.96

Figure 6A – tabular results – SM vs CL

	Unpaired t test<br>Tabular results	
1	Table Analyzed	screen lipids + drugs_for new figure 5A
2		
3	Column H	SM
4	vs.	vs.
5	Column G	CL
6		
7	**Unpaired t test**	
8	P value	0.5756
9	P value summary	ns
10	Significantly different (P < 0.05)?	No
11	One- or two-tailed P value?	Two-tailed
12	t, df	t=0.6086, df=4
13		
14	**How big is the difference?**	
15	Mean of column G	10.40
16	Mean of column H	11.30
17	Difference between means (H - G) ± SEM	0.9000 ± 1.479
18	95% confidence interval	-3.206 to 5.006
19	R squared (eta squared)	0.08476
20		
21	**F test to compare variances**	
22	F, DFn, Dfd	2.023, 2, 2
23	P value	0.6616
24	P value summary	ns
25	Significantly different (P < 0.05)?	No
26		
27	**Data analyzed**	
28	Sample size, column G	3
29	Sample size, column H	3

Figure 6A – descriptive statistics – SM vs CL

	Unpaired t test<br>Descriptive statistics	CL	SM
1	Number of values	3	3
2			
3	Minimum	8.600	10.00
4	25% Percentile	8.600	10.00
5	Median	9.900	11.00
6	75% Percentile	12.70	12.90
7	Maximum	12.70	12.90
8			
9	Mean	10.40	11.30
10	Std. Deviation	2.095	1.473
11	Std. Error of Mean	1.210	0.8505
12			
13	Lower 95% CI	5.195	7.641
14	Upper 95% CI	15.60	14.96

Figure 6A – tabular results – SM vs Lyso-PC

Unpaired t test		
1	Table Analyzed	screen drugs
2		
3	Column E	SM
4	vs.	vs.
5	Column B	Lyso-PC
6		
7	**Unpaired t test**	
8	P value	0.0002
9	P value summary	***
10	Significantly different (P < 0.05)?	Yes
11	One- or two-tailed P value?	Two-tailed
12	t, df	t=13.72, df=4
13		
14	**How big is the difference?**	
15	Mean of column B	36.40
16	Mean of column E	11.30
17	Difference between means (E - B) ± SEM	-25.10 ± 1.829
18	95% confidence interval	-30.18 to -20.02
19	R squared (eta squared)	0.9792
20		
21	**F test to compare variances**	
22	F, DFn, Dfd	3.627, 2, 2
23	P value	0.4323
24	P value summary	ns
25	Significantly different (P < 0.05)?	No

Figure 6A – tabular results – SM vs edelfosine

Unpaired t test		
1	Table Analyzed	screen drugs
2		
3	Column E	SM
4	vs.	vs.
5	Column C	Edelfosine
6		
7	**Unpaired t test**	
8	P value	0.0071
9	P value summary	**
10	Significantly different (P < 0.05)?	Yes
11	One- or two-tailed P value?	Two-tailed
12	t, df	t=5.068, df=4
13		
14	**How big is the difference?**	
15	Mean of column C	21.77
16	Mean of column E	11.30
17	Difference between means (E - C) ± SEM	-10.47 ± 2.065
18	95% confidence interval	-16.20 to -4.733
19	R squared (eta squared)	0.8653
20		
21	**F test to compare variances**	
22	F, DFn, Dfd	4.896, 2, 2
23	P value	0.3392
24	P value summary	ns
25	Significantly different (P < 0.05)?	No

Figure 6A – tabular results – SM vs miltefosine

Unpaired t test		
1	Table Analyzed	screen drugs
2		
3	Column E	SM
4	vs.	vs.
5	Column D	Miltefosine
6		
7	**Unpaired t test**	
8	P value	0.0177
9	P value summary	*
10	Significantly different (P < 0.05)?	Yes
11	One- or two-tailed P value?	Two-tailed
12	t, df	t=3.887, df=4
13		
14	**How big is the difference?**	
15	Mean of column D	19.33
16	Mean of column E	11.30
17	Difference between means (E - D) ± SEM	-8.033 ± 2.067
18	95% confidence interval	-13.77 to -2.295
19	R squared (eta squared)	0.7907
20		
21	**F test to compare variances**	
22	F, DFn, Dfd	4.905, 2, 2
23	P value	0.3387
24	P value summary	ns
25	Significantly different (P < 0.05)?	No
