# Supplementary material for: Autoinhibition and regulation by phosphoinositides of ATP8B1, a human lipid flippase associated with intrahepatic cholestatic disorders
Source: eLife. 2022 Apr 13;11:e75272. doi: 10.7554/eLife.75272 (PMC9045818; doi:10.7554/eLife.75272)
Supplement: Figure 6—source data 2. [file elife-75272-fig6-data2.pdf]

Figure 6B – source data

	Group A	Group B	Group C	Group D	Group E	Group F	Group G	Group H
	$PI(4,5)P_2$	$PI(3,5)P_2$	$PI(3,4)P_2$	PI(5)P	PI(4)P	PI(3)P	PI	$PI(3,4,5)P_3$
1	94.5	88.6	81.2	35.7	38.8	33.3	4.5	145.9
2	103.9	72.5	77.7	37.0	39.8	36.4	8.2	155.8
3	101.6	86.7	80.4	36.6	44.0	34.2	6.9	142.4
