# Supplementary material for: Autoinhibition and regulation by phosphoinositides of ATP8B1, a human lipid flippase associated with intrahepatic cholestatic disorders
Source: eLife. 2022 Apr 13;11:e75272. doi: 10.7554/eLife.75272 (PMC9045818; doi:10.7554/eLife.75272)
Supplement: Figure 6—source data 3. [file elife-75272-fig6-data3.pdf]

Figure 6C – source data

Table format: XY		X	Group A				Group B				Group C			
		PIP/DDM (mole/mole)	PI4P (μmol/min)				PI(4,5)P2 (μmol/min)				PI(3,4,5)P3 (μmol/min)			
		X	A:Y1	A:Y2	A:Y3	A:Y4	B:Y1	B:Y2	B:Y3	B:Y4	C:Y1	C:Y2	C:Y3	C:Y4
1	Title	0.0000	0.0	0.0	0.0	0.0								
2	Title	0.0232	94.1	128.9	111.5	106.3								
3	Title	0.0170	67.9	90.6	87.1	78.4								
4	Title	0.0130	62.7	76.6	64.5	64.5								
5	Title	0.0110	52.3	55.7	54.0	50.5								
6	Title	0.0080	48.8	50.5	38.3	45.3								
7	Title	0.0060	34.8	38.3	36.6	34.8								
8	Title	0.0050	33.1	26.1	24.4	31.4								
9	Title	0.0040	31.4	26.1	22.6	26.1								
10	Title	0.0030	19.2	24.4	13.9	13.9								
11	Title	0.0235						139.4	149.8	155.0				
12	Title	0.0170						125.4	121.9					
13	Title	0.0130						94.1	104.5	111.5				
14	Title	0.0110						80.1	95.8					
15	Title	0.0080						64.5	74.9	92.3				
16	Title	0.0060					71.4	55.7	71.4					
17	Title	0.0050					62.7	48.8	67.9	66.2				
18	Title	0.0040						38.3	55.7	55.7				
19	Title	0.0030					48.8	31.4	22.6	45.3				
20	Title	0.0219					170.7							
21	Title	0.0160					139.4							
22	Title	0.0120					123.7							
23	Title	0.0100					104.5							
24	Title	0.0070					83.6							
25	Title	0.0020					36.6							
26	Title	0.0237									156.8	146.3	169.0	
27	Title	0.0170									153.3			
28	Title	0.0130									151.5	151.5	148.1	
29	Title	0.0110									149.8			
30	Title	0.0080									144.6	139.4	142.8	
31	Title	0.0060									142.8			
32	Title	0.0050									137.6	123.7	130.6	
33	Title	0.0040									132.4	115.0	123.7	
34	Title	0.0026										101.0	106.3	
35	Title	0.0017										85.4	87.1	
36	Title	0.0012										76.6	80.1	
37	Title	0.0009										66.2	67.9	

Figure 6C – fit

Nonlin fit<br>Table of results		A<br>PI4P (µmol/min)	B<br>PI(4,5)P2 (µmol/min)	C<br>PI(3,4,5)P3 (µmol/min)
		Y	Y	Y
1	**Michaelis-Menten**			
2	**Best-fit values**			
3	Vmax	466.4	269.7	167.7
4	Km	0.07731	0.01834	0.001445
5	**Std. Error**			
6	Vmax	126.5	27.55	2.423
7	Km	0.02527	0.003189	9.109e-005
8	**95% CI (asymptotic)**			
9	Vmax	210.2 to 722.5	213.5 to 325.9	162.7 to 172.7
10	Km	0.02616 to 0.1285	0.01184 to 0.02485	0.001257 to 0.001633
11	**Goodness of Fit**			
12	Degrees of Freedom	38	31	24
13	R squared	0.9596	0.9185	0.9693
14	Sum of Squares	1592	3967	715.3
15	Sy.x	6.473	11.31	5.459
16	**Constraints**			
17	Km	Km > 0	Km > 0	Km > 0
18				
19	**Number of points**			
20	# of X values	40	100	148
21	# Y values analyzed	40	33	26
