# Supplementary material for: Autoinhibition and regulation by phosphoinositides of ATP8B1, a human lipid flippase associated with intrahepatic cholestatic disorders
Source: eLife. 2022 Apr 13;11:e75272. doi: 10.7554/eLife.75272 (PMC9045818; doi:10.7554/eLife.75272)
Supplement: Figure 6—source data 4. [file elife-75272-fig6-data4.pdf]

Figure 6D – source data – Vm

Table format: Grouped	Group A PI(4)P			Group B $PI(4,5)P_2$			Group C $PI(3,4,5)P_3$		
	Mean	SD	N	Mean	SD	N	Mean	SD	N
1 Vm	1.000	0.070	36	1.160	0.1080	33	0.78	0.023	26

Figure 6D – source data – Km

Table format: Grouped	Group A PI(4)P			Group B $PI(4,5)P_2$			Group C $PI(3,4,5)P_3$		
	Mean	SD	N	Mean	SD	N	Mean	SD	N
1 Km	1.000	0.070	36	1.820	0.1700	33	21.70000	0.65000	26
