# Supplementary material for: Autoinhibition and regulation by phosphoinositides of ATP8B1, a human lipid flippase associated with intrahepatic cholestatic disorders
Source: eLife. 2022 Apr 13;11:e75272. doi: 10.7554/eLife.75272 (PMC9045818; doi:10.7554/eLife.75272)
Supplement: Figure 6—figure supplement 1—source data 1. [file elife-75272-fig6-figsupp1-data1.pdf]

Figure 6 – figure supplement 1 – source data

Table format: XY		X	Group A				Group B				Group C			
		1/[S]	1/v (PI4P)				1/v (PI4,5P2)				1/v (PI3,4,5)P3			
		X	A:Y1	A:Y2	A:Y3	A:Y4	B:Y1	B:Y2	B:Y3	B:Y4	C:Y1	C:Y2	C:Y3	C:Y4
1	Title	43.2	0.185185	0.135135	0.156250	0.163934								
2	Title	59.8	0.256410	0.192308	0.200000	0.222222								
3	Title	76.5	0.277778	0.227273	0.270270	0.270270								
4	Title	93.2	0.333333	0.312500	0.322581	0.344828								
5	Title	126.6	0.357143	0.344828	0.454546	0.384615								
6	Title	159.9	0.500000	0.454546	0.476191	0.500000								
7	Title	193.3	0.526316	0.666667	0.714286	0.555556								
8	Title	260.0	0.555556	0.666667	0.769231	0.666667								
9	Title	393.4	0.909091	0.714286	1.250000	1.250000								
10	Title	42.6						0.125000	0.116279	0.112360				
11	Title	59.1						0.138889	0.142857					
12	Title	75.6						0.185185	0.166667	0.156250				
13	Title	92.0						0.217391	0.181818					
14	Title	124.9						0.270270	0.232558	0.188679				
15	Title	157.8						0.312500	0.243902					
16	Title	190.8						0.357143	0.256410	0.263158				
17	Title	256.6						0.454546	0.312500	0.312500				
18	Title	388.2						0.555556	0.769231	0.384615				
19	Title	45.6					0.102041							
20	Title	64.3					0.125000							
21	Title	83.1					0.140845							
22	Title	101.8					0.166667							
23	Title	139.3					0.208333							
24	Title	176.8					0.243902							
25	Title	214.3					0.277778							
26	Title	289.3					0.357143							
27	Title	439.3					0.476191							
28	Title	42.2									0.111111			
29	Title	58.5									0.113636			
30	Title	74.9									0.114943			
31	Title	91.2									0.116279			
32	Title	123.8									0.120482			
33	Title	156.4									0.121951			
34	Title	189.1									0.126582			
35	Title	254.3									0.131579			
36	Title	42.2										0.119048	0.103093	
37	Title	74.9										0.114943	0.117647	
38	Title	123.8										0.125000	0.121951	
39	Title	189.1										0.140845	0.133333	
40	Title	254.3										0.151515	0.140845	
41	Title	384.8										0.172414	0.163934	
42	Title	580.6										0.204082	0.200000	
43	Title	841.6										0.227273	0.217391	
44	Title	1102.6										0.263158	0.256410	

Figure 6 – figure supplement 1 – fit – PI4P

	Simple linear regression	A
		1/v (PI4P)
		Y
1	Best-fit values	
2	Slope	0.002420 ± 0.0001483
3	Y-intercept when X=0.0	0.08272 ± 0.02803
4	X-intercept when Y=0.0	-34.18
5	1/slope	413.2
6	95% Confidence Intervals	
7	Slope	0.002118 to 0.002722
8	Y-intercept when X=0.0	0.02572 to 0.1397
9	X-intercept when Y=0.0	-64.75 to -9.628
10	Goodness of Fit	
11	R square	0.8867
12	Sy.x	0.09461
13	Is slope significantly non-zero?	
14	F	266.1
15	DFn, DFd	1.000, 34.00
16	P value	< 0.0001
17	Deviation from zero?	Significant
18	Data	
19	Number of X values	9
20	Maximum number of Y replicates	4
21	Total number of values	36
22	Number of missing values	140
23		
24	Equation	Y = 0.002420*X + 0.08272

Figure 6 – figure supplement 1 – fit – PI(4,5)P2

	Simple linear regression	A
		1/v (PI4,5P2)
		Y
1	Best-fit values	
2	Slope	0.001146 ± 0.0001008
3	Y-intercept when X=0.0	0.07146 ± 0.02000
4	X-intercept when Y=0.0	-62.37
5	1/slope	872.8
6	95% Confidence Intervals	
7	Slope	0.0009402 to 0.001351
8	Y-intercept when X=0.0	0.03067 to 0.1123
9	X-intercept when Y=0.0	-116.5 to -23.25
10	Goodness of Fit	
11	R square	0.8065
12	Sy.x	0.06474
13	Is slope significantly non-zero?	
14	F	129.2
15	DFn, DFd	1.000, 31.00
16	P value	< 0.0001
17	Deviation from zero?	Significant
18	Data	
19	Number of X values	18
20	Maximum number of Y replicates	3
21	Total number of values	33
22	Number of missing values	143
23		
24	Equation	Y = 0.001146*X + 0.07146

Figure 6 – figure supplement 1 – fit – PI(3,4,5)P3

Simple linear regression		A
		1/v (PI3,4,5)P3
		Y
1	Best-fit values	
2	Slope	0.0001436 ± 4.204e-006
3	Y-intercept when X=0.0	0.1060 ± 0.001884
4	X-intercept when Y=0.0	-738.2
5	1/slope	6966
6	95% Confidence Intervals	
7	Slope	0.0001349 to 0.0001522
8	Y-intercept when X=0.0	0.1021 to 0.1099
9	X-intercept when Y=0.0	-808.8 to -675.4
10	Goodness of Fit	
11	R square	0.9798
12	Sy.x	0.006845
13	Is slope significantly non-zero?	
14	F	1166
15	DFn, DFd	1.000, 24.00
16	P value	< 0.0001
17	Deviation from zero?	Significant
18	Data	
19	Number of X values	17
20	Maximum number of Y replicates	2
21	Total number of values	26
22	Number of missing values	150
23		
24	Equation	Y = 0.0001436*X + 0.1060
