# Supplementary material for: Autoinhibition and regulation by phosphoinositides of ATP8B1, a human lipid flippase associated with intrahepatic cholestatic disorders
Source: eLife. 2022 Apr 13;11:e75272. doi: 10.7554/eLife.75272 (PMC9045818; doi:10.7554/eLife.75272)
Supplement: Figure 6—figure supplement 2—source data 1. [file elife-75272-fig6-figsupp2-data1.pdf]

Figure 6 – figure supplement 2B – source data

		X	Group A				Group B			
		Ve, mL	Protein, pmole				14C-DDM, pmole			
		X	A:Y1	A:Y2	A:Y3	A:Y4	B:Y1	B:Y2	B:Y3	B:Y4
1	T	5.25	33.30	29.00			29263.0			
2	T	5.50	13.60	24.70			20455.0			
3	T	5.75	25.90	37.00			20057.0			
4	T	6.00	31.50	38.90			8531.0			
5	T	6.25	30.80	25.30			8462.0			
6	T	6.50	64.20	111.10			28675.0			
7	T	6.75	121.60	98.80			23639.0			
8	T	7.00	82.10	54.90			18793.0			
9	T	7.25	40.10	33.30			7199.0			
10	T	7.50	22.80	29.00			9016.0			
11	T	7.75	24.70	16.10			1696.0			
12	T	8.00	6.20	21.00			0.0			
13	T	8.25	6.80	0.00			3634.0			
14	T	8.50	0.00	0.60			10383.0			
15	T	8.75	9.90	7.40			11871.0			
16	T	9.00	19.10	0.00			118782.0			
17	T	9.25	13.00	3.70			123611.0			
18	T	9.50	6.80	0.00			32257.0			
19	T	9.75	6.20	1.30			3755.0			
20	T	10.00	19.10	17.30			-1782.0			
